# Beyond the Surface: The Critical Role of Interdisciplinary Collaboration in Managing Parastomal Hernias

**DOI:** 10.3389/jaws.2025.15255

**Published:** 2025-09-29

**Authors:** Marianne Krogsgaard, Frederik Helgstrand

**Affiliations:** ^1^ Department of Surgery and Center for Surgical Science, Zealand University Hospital, Koege, Denmark; ^2^ Department of People and Technology, Roskilde University, Roskilde, Denmark; ^3^ Department of Clinical Medicine, University of Copenhagen, Copenhagen, Denmark

**Keywords:** parastomal hernia, surgical management, non-operative management, interdisciplinary collaboration, patient-centered care

## Introduction

The creation of a stoma inherently results in an abdominal wall defect. Parastomal hernias are defined as an abnormal protrusion of abdominal contents through the abdominal wall defect [1]. This condition affects up to 50% of patients, depending on the stoma type and follow-up duration [2]. To date, surgical management of this condition has been challenging.

Patients may experience discomfort, stoma dysfunction, appliance leakage, peristomal skin problems, and psychosocial distress. While not all hernias progress to severe complications, many result in chronic lifestyle limitations.

Although often regarded as a cosmetic issue, parastomal hernias have deep functional and psychological ramifications that can impair a patient’s quality of life significantly [3, 4]. Management is rarely linear and demands a nuanced, individualised approach. Central to successful outcomes is the interdisciplinary collaboration—especially between surgeons and nurses—which ensures not only technical care but also support, information and education that are known predictors of better outcomes [5].

In this article, we promote a patient-centred, interdisciplinary approach to the patient presenting with a parastomal bulge, focusing on the patient’s key problems and concerns to improve outcomes.

## Nurses and Surgeons: Complementary Roles

Nurses, particularly stoma care nurses, play a key role in identifying bulging and early symptoms, ensuring proper stoma appliance fitting, managing peristomal skin issues, and providing crucial education and emotional support. Surgeons offer diagnostic evaluation, determine operative eligibility, and perform complex repairs. Together, they form a synergistic team that can adapt treatment plans to each patient’s evolving needs.

## Trajectory of Parastomal Hernia Management: A Dynamic Pathway

Parastomal hernia care follows a trajectory rather than a fixed protocol. Only a minority of patients are likely to undergo surgery, with up to 80% of patients either treated by stoma care nurses or handling the parastomal hernia themselves [6]. Many cases begin with observation and non-operative management, especially if the hernia is asymptomatic or minimally symptomatic. The trajectory often shifts in response to functional decline, quality-of-life impacts, or the emergence of complications such as leakage and pain. Surgical treatments are often high-risk procedures with up to 50% recurrence rates in the long term [7]. Under this condition, a parastomal hernia surgeon may be considered a ‘quality of life specialist’, treating a bulging condition that is highly likely to recur. Thus, unless there is an absolute indication for surgery, initially non-operative management by a stoma care nurse should always be considered standard of care.

## Aligning Expectations: Understanding what Matters to Patients

**FIGURE 1:**
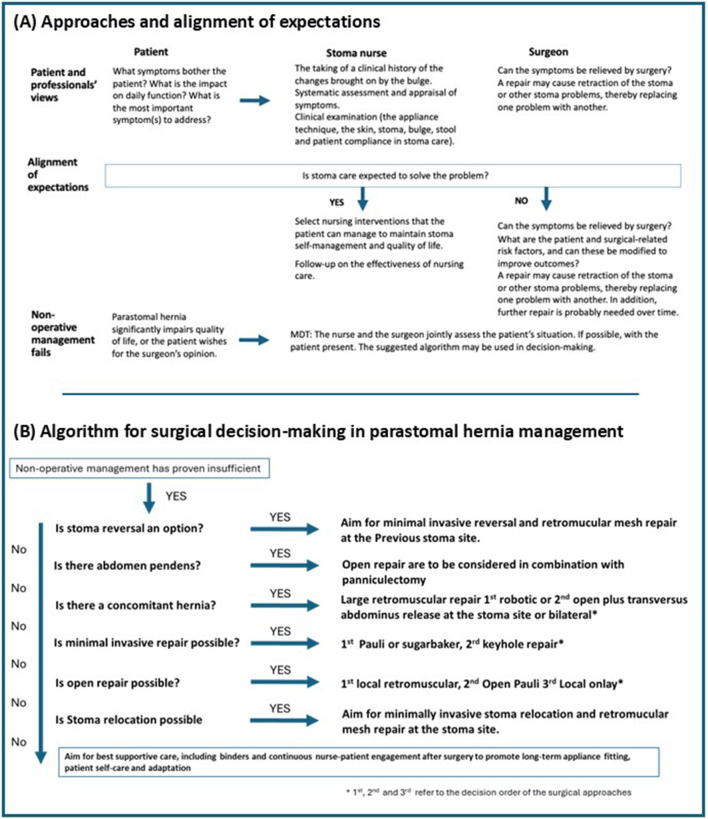
Suggested clinical pathway for parastomal hernia management. **(A)** Approaches and alignment of expectations. **(B)** Algorithm for surgical decision-making in parastomal hernia management.

Although the patient finds the parastomal hernia difficult to live with, their bother and symptoms may not align neatly with medical classifications of “severity”. Unpredictability of appliance leaks, feeling restricted in public settings, or pain during movement are of great importance in everyday life. To others, the hernias’ physical appearance or fear of bulge enlargement may be a key concern [4, 8]. Likewise, patients have differing profiles; some may see surgical repair as the only hope for cure, while others may find surgical repair too risky [9]. Therefore, exploring and understanding the patient’s perspective and most important problems related to the hernia is key to providing patients with the best functional results [10]. Figure 1A provides detailed information on approaches and expectation alignment.

Nurses, through sustained relationships, often serve as advocates who translate patient concerns into clinical language. When surgeons and nurses engage in shared decision-making with the patient, the team can better set realistic expectations, discuss treatment trade-offs, and develop care plans that address what patients value, such as regaining independence or returning to work. Likewise, joint agreement and understanding of the overall patient trajectory, information content and referral pathways are ways to preserve and improve patients’ trust in healthcare professionals, which may be specifically needed in a field lacking high-quality treatment options [9].

## Discussion

### Operative vs Non-operative Treatment Options

#### Non-Operative Management, Nursing Care

Depending on the presentation and symptoms of the parastomal hernia, the stoma care nurse has various options to relieve patients’ symptoms. A single intervention can address multiple symptoms, while multiple interventions might be needed for a single symptom [11].

Support garment: Is the most frequently used approach that can manage symptoms such as bearing down, poor wear time of appliances, dragging sensation, altered bodily appearance, irregular stool and fear of being physically active. They provide compression, reduce protrusion and may improve comfort and security. Support garments come in various forms (with or without a hole for the stoma) and compression levels, including belts, briefs, tubes and sportswear. To choose the right garment, the nurse assesses patient expectations, symptoms, lifestyle and comorbidities [12, 13]. Standard garments are preferred while bespoke ones are more rigid and expensive [12, 13]. Practical testing, tailored information, and follow-up are key enablers of belt adherence [12]. The stoma care nurse is aware of the risk of garments inducing symptoms such as pressure ulcers, leakage and skin irritation [11]. Garments may not be a solution if the parastomal hernia is irreducible due to the risk of pressure ulcers [11, 12].

Management of ostomy products: Nurses address issues such as poor wear time, skin problems, and leakage by adjusting patients’ appliances [11]. Stoma care nurses regularly remeasure the stoma as its size, height and shape change with a parastomal hernia. Over time, the bulge may enlarge and alter shape, stretching the skin, making it thin and fragile. For the patient, the pouching procedure may become increasingly complex [4, 14], necessitating the use of accessories and a change of stoma product. Nurses often instruct patients on new fitting procedures and the use of a mirror due to impaired visibility of the stoma.

Irrigation and Regulation of stool: Irrigation difficulties, diarrhoea, constipation and irregular stool may come with the hernia [4, 11]. Thus, stoma care nurses assess the patient’s diet and functional output, advise about dietary and fluid regulation and proper exercise, and prescribe bulk laxatives or stool softeners. Some patients may continue to irrigate their colostomy while others are advised to stop if the water can no longer pass in or out [11].

Nursing care also concerns disguise and intimacy, helping patients find ways to hide the bulge. Other elements of nursing care include providing information and education on physical activity, offering advice and education on how to assess signs and symptoms of strangulation and delivering psychological support and education on how to live with the parastomal hernia [11]. Stoma care nurses offer follow-up and reassessment, align care with the primary sector and ensure involvement of the surgeon when needed.

#### Operative Management

Surgical repair remains technically challenging and is associated with recurrence rates of up to 30%–50%, as well as high risk for severe complications [15]. However, if non-operative management proves insufficient and the patient’s symptoms and impaired quality of life are deemed to outweigh the risk for surgery, a repair should be offered. A suggested algorithm, based on current literature and expert opinion, for the surgical management of parastomal hernias is outlined in Figure 1B [2, 16–19]. Patients with parastomal hernias are very heterogeneous, and most often the surgical approach must be tailored to the individual patient to align expectations and to achieve the best possible result. Thus, although a mesh-reinforced repair is considered the gold standard to avoid recurrences, there are situations where sutured repair may be the best option.

Certain conditions demand operative intervention: Incarceration, strangulation and obstructions are life-threatening conditions that need emergent surgery. In those situations, less is more, and the focus should be to save the patient, and often, a sutured repair of the defect is enough. A more durable mesh repair can be planned later.

Fortunately, 90 percent of patients undergo surgery in an elective setting. Those patients are characterised mainly by feeling socially restricted, lifestyle limitations, severe pain, intermittent obstruction and stoma dysfunction such as poor appliance fit, frequent leakage, or prolapse that cannot be managed conservatively.

Since many patients will have recurrence, and especially young patients are likely to undergo another repair in the future, the best way to treat parastomal hernias is to do stoma reversal. For many patients, this could be a viable option [6]. If stoma reversal is impossible, it is important that the surgical strategy does not unnecessarily compromise any future operations. For the same reasons, stoma relocation, where the stoma is relocated to another abdominal quadrant, combined with hernia repair, should be considered as the last option [6]. This is because a high number of those patients will end up with a new parastomal hernia as well as a hernia recurrence at the previous stoma site [20, 21].

Another aspect to consider is the patients problem. The treatment of a patient with a bulge and leakage may not necessarily be the same as the treatment for a patient with symptoms of incarceration.

The patients body shape should also be considered. For instance, many patients may benefit from resection of excessive skin. However, almost all patients will continue to have an altered body shape after surgery, and many patients may end up with a retracted stoma that may provide skin and leakage problems.

In most situations, a synthetic mesh is preferred. Although there is no existing evidence, it is suggested to consider absorbable synthetic or biologic meshes and to avoid intraperitoneal mesh positions in patients with a history of fistulating Crohn’s disease.

When deciding on elective surgery, it is important to aim for minimally invasive surgery and address patient-related risk factors to reduce the risk of complications. Prehabilitation, such as weight loss, smoking cessation and optimal treatment of other co-morbidities, is often required to obtain safe surgery.

### Benefits of Interdisciplinary Collaboration

As described, parastomal hernia patients are very heterogeneous, and the treatment is complex. Therefore, interdisciplinary collaboration and alignment of expectations are essential to achieve improvements. Patients will benefit from interdisciplinary collaboration throughout their often long trajectory. This evolving path underscores the importance of ongoing interdisciplinary communication and patient involvement.

### Barriers and Solutions

Despite evidence for collaborative success, barriers such as siloed care, lack of shared protocols, and underutilization of nursing input persist.

Hospitals and surgical departments should:

• Implement multidisciplinary team (MDT) meetings for complex stoma cases
• Develop standardised care pathways that embed nursing evaluations into surgical assessments
• Promote cross-training and joint education programs on parastomal hernia management

## Conclusion

Parastomal hernias are complex, dynamic conditions that demand more than episodic surgical interventions. From conservative nursing care to surgical repair, the entire management pathway is strengthened when nurses and surgeons operate as equal partners. Most importantly, care must center around the patient, addressing what matters most to them, aligning expectations, and adapting to their lived experience. Interdisciplinary collaboration is not an option; it’s a necessity for delivering care that is safe, effective, and humane.
